# Factors associated with hospital anxiety and depression among ICU survivors: a cross sectional study

**DOI:** 10.1186/2197-425X-3-S1-A369

**Published:** 2015-10-01

**Authors:** R Rosa, A Ascoli, W Rutzen, L Madeira, M Falavigna, C Robinson, C Nascimento, P Balzano, P Morandi, V Souto, M Moreira, M Mutlaq, K Lima, MC Souza, R Ribeiro, J Maccari, C Almeida, RPD Oliveira, C Teixeira

**Affiliations:** Moinhos de Vento Hospital, Porto Alegre, Brazil

## Introduction

Intensive care unit (ICU) survivors are at risk of developing a number of psychological problems. However, there is currently no agreement on how to determine the individual patient's risk for psychological dysfunction after critical illness.

## Objectives

This study sought to evaluate factors associated with hospital anxiety and depression in adult ICU survivors.

## Methods

A multicenter cross sectional study was conducted in Southern Brazil between May 2014 and March 2015. The Hospital Anxiety and Depression Scale (HADS) were applied in all consecutive ICU survivors within the first 72 hours after discharge from ICU. A stepwise multiple linear regression was performed to identify factors associated with symptoms of anxiety and depression among ICU survivors.

## Results

In total, 101 patients (42% men) were evaluated. The mean age and APACHE-II score were 62.9 years (SD 17.0) and 13.3 points (SD 5.2), respectively. The mean ICU length of stay was 7.6 days (SD 7.6). The mean HADS scale score of the study population was 6.4 points (SD 4.2). According to multiple linear regression analysis, adjusted for gender and previous diagnosis of mood disorder, renal replacement therapy (RRT) need during ICU stay (β=+2.28, *p* = 0.006), admission in multi-bed ICU room (β=+1.72, *p* = 0.03), and previous diagnosis of mood disorder (β=+2.75, *p* = 0.005) were positively associated with symptoms of anxiety after ICU discharge. In a second multiple linear regression model, adjusted for gender and previous diagnosis of mood disorder, RRT during ICU stay (β=+2.44, *p* = 0.008), admission in multi-bed ICU room (β=+1.56, *p* = 0.02) and previous diagnosis of mood disorder (β=+2.03, *p* = 0.03) were positively associated with symptoms of depression after ICU discharge.

## Conclusions

Our study suggests a complex etiology for the development of psychological disorders after ICU discharge, as both patient characteristics, severity of disease, and ICU-related variables represented risk factors for psychological dysfunction after critical illness.
